# Supplementary material for: Predicting Neoadjuvant Chemotherapy Response in Triple-Negative Breast Cancer Using Pre-Treatment Histopathologic Images
Source: Cancers (Basel). 2025 Jul 22;17(15):2423. doi: 10.3390/cancers17152423 (PMC12346615; doi:10.3390/cancers17152423)
Supplement: Supplementary file 1 [file cancers-17-02423-s001.zip › cancers-3685252-supplementary.pdf]

Supplementary Materials

Supplementary Table S1. Distribution of patient cohorts used in this study, including the number of cases with pathological complete response (pCR) and pathological incomplete response (non-pCR), for both the in-house OSU Wexner Medical Center cohort and the independent MD Anderson Cancer Center cohort. For the in-house cohort, data were split for five-fold cross-validation using an average ratio of 70% for training, 10% for validation, and 20% for testing.

Cohorts	Format	Magnification	Average Dimension	pCR cases	Non-pCR cases	Total
OSU-Wexner Medical Center (in-house)	.svs	40x	90k x 90k	81	93	174
MD-Anderson Cancer Center (independent)	.svs	40x	90k x 90k	12	18	30

Supplementary Table S2. List of core software libraries and packages used in the study, along with their respective version numbers to ensure reproducibility.

Library	Version	Library	Version	Library	Version
H5py	3.13.0	Wandb	0.19.8	Torch	2.6.0+cu118
Numpy	1.24.2	Dill	0.3.9	Torchvision	0.21.0+cu118
Pathlib	1.0.1	Matplotlib	3.10.1	Trident	0.0.5
Python	3.10.9	Anaconda	24.1.2	Pandas	1.4.4
OpenSlide	3.4.1			Scikit-learn	1.3

Supplementary Table S3. Grid search ranges for key hyperparameters explored during model selection. Each combination of values was used to train models under a standardized protocol. The search aimed to identify optimal configurations for attention-based MIL model.

Hyperparameter	Values Explored	Hyperparameter	Version
Learning rate	{0.0001, 0.001, 0.01}	Optimizer	{SGD, Adam}
Weight decay	{0.0001, 0.001}	Patch Encoder	UNI_V1
Attention heads	{1, 2}		

Supplementary Table S4. Established hyperparameters for attention-based MIL model.

Hyperparameter	Value	Hyperparameter	Version
Epochs	1024	Patch Encoder	UNI_V1
Learning rate	0.0001	Attention heads	1
Model Architecture	ABMIL	Patience for early stopping	50 (epochs)
Optimizer	SGD	Weight decay	0.001
Patch size	512 x 512	Magnification	40x
# of fully connected layers	1	Dimension of fully connected layers	1024
Dropout	0.1		

Supplementary Table S5. Grid search ranges for key hyperparameters explored during ML model selection.

Hyperparameter	Values Explored	Hyperparameter	Version

Number of Trees	{10, 20, 30, 40, 50}	Optimizer	{SGD, Adam}
Depth of Trees	{100, 150, 200}	Patch Encoder	UNI_V1
C	{0.001, 0.01, 0.1, 1, 10, 100, 1000}	Gamma	{0.001, 0.01, 0.1, 1, 10, 100, 1000}

Supplementary Table S6. Established hyperparameters for MLs.

MLs	Value
RF (Randon Forest)	number of trees = 50, depth of trees = 100
KNN	n_neighbors = 5, algorithm=KDTree
SVM (RBF)	kernel = rbf , C = 1.0, gamma = scale
SVM (Linear)	kernel = linear , C = 1.0
Logistic Regression	max_iteration = 1000
Decision Tree	max_depth = 5
Gradient Boosting	n_estimators = 100, learning_rate = 0.1, max_depth = 3
Naïve Bayes	GaussianNB
Linear Discriminant Analysis	solver = svd, tol = 0.0001
Quadratic Discriminant Analysis	tol = 0.0001
